# SARS-CoV-2, long COVID, prion disease and neurodegeneration

**DOI:** 10.3389/fnins.2022.1002770

**Published:** 2022-09-27

**Authors:** Yuhai Zhao, Vivian R. Jaber, Walter J. Lukiw

**Affiliations:** ^1^Department of Cell Biology and Anatomy, Louisiana State University Health Sciences Center, New Orleans, LA, United States; ^2^LSU Neuroscience Center, LSU Health Sciences Center, New Orleans, LA, United States; ^3^Department of Ophthalmology, LSU Health Sciences Center, New Orleans, LA, United States; ^4^Department of Neurology, LSU Health Sciences Center, New Orleans, LA, United States

**Keywords:** Creutzfeldt-Jacob disease (CJD), long COVID-19, ‘S1’ spike protein, SARS-CoV-2, prion disease (PrD), Alzheimer's disease (AD), miRNA-146a, miRNA-155

## Introduction

On the last day of the year 2019 a novel *Betacoronavirus* (2019-nCov), now known as severe acute respiratory syndrome coronavirus 2 (SARS-CoV-2), and causing the highly transmissible and lethal pneumonia COVID-19 was first reported in Wuhan, Hubei Province in Central China (Huang et al., 2020; Fu et al., 2022; Lu and Sun, 2022). Since then ongoing research and long-term studies of the sequelae of SARS-CoV-2 infection have indicated that post-infection, recovery from COVID-19 and/or COVID-19 aftermath is associated with long-term physiological and neurological deficits known generically as “long COVID” (Roy et al., 2021; Ahmad et al., 2022; Baazaoui and Iqbal, 2022). Multiple independent epidemiological and clinical studies further indicate that SARS-CoV-2 infection and “long COVID” strongly correlate with the onset of progressive neurological disturbances that include Alzheimer's disease (AD), prion disease (PrD) and other neurodegenerative disorders. These are apparent: **(i)** especially in aged and/or senile COVID-19 patients; **(ii)** in patients experiencing overlapping or inter-current illnesses that include heart disease, diabetes, hypertension, neuropsychiatric and other age-related neurological disorders; and **(iii)** in those COVID-19 patients who have experienced a particularly virulent and/or a near fatal episode of SARS-CoV-2 infection (Farheen et al., 2021; Flud et al., 2022; Fu et al., 2022). Conversely, increasing numbers of epidemiological studies suggest that elderly people with neurological deficits commonly observed in AD are highly vulnerable to SARS-CoV-2 infection, and especially the development of more severe forms of COVID-19 disease (Chiricosta et al., 2021; Hsu et al., 2021; Fu et al., 2022). The recent finding that the SARS-CoV-2 “S1” spike protein essential for viral infectivity contains prion-like domains associated with immune-evasion and the promotion of protein aggregation and aggregate “seeding” is particularly intriguing (Baazaoui and Iqbal, 2022; Bernardini et al., 2022; Tetz and Tetz, 2022). Based on these and other very recent findings this “Opinion” paper will: **(i)** address our current understanding of the emerging role of SARS-CoV-2 infection with “long COVID” with special reference to AD and PrD; **(ii)** will review the latest findings of the SARS-CoV-2 “S1” spike protein and its preferred interaction with the ubiquitous angiotensin converting enzyme-2 (ACE2) receptor (ACE2R); and **(iii)** will highlight the interplay of the molecular biology and neuropathology of SARS-CoV-2 with the unusual and immune-evasive character of prion neurobiology, AD and PrD.

## SARS-CoV-2, “long COVID” and neurological disease

The SARS-CoV-2 virus that causes COVID-19 disease is a highly contagious pathogen that continues to impact human health around the globe and is responsible for one of the worst pandemics in recorded human history. Of the ~600 million people that have been infected about half of all COVID-19 patients exhibit the symptomology of “long COVID” and many experience some type of lingering neurological complications including, prominently, “brain fog,” confusion, impaired consciousness, deficits in cognition and memory, encephalopathy, encephalitis and/or cerebrovascular deficits (Mao et al., 2020; Roy et al., 2021; Ahmad et al., 2022; Baazaoui and Iqbal, 2022; Visco et al., 2022; https://www.worldometers.info/coronavirus/coronavirus-death-toll/; https://www.science.org/content/article/what-causes-long-covid-three-leading-theories?cookieSet=1; https://www.forbes.com/sites/joshuacohen/2022/06/22/dutch-research-on-long-covid-shows-50-of-study-partic-ipants-have-1-or-more-symptoms-3-months-after-becoming-infected-with-coronavirus/?sh$=$45228b705a6a; last accessed 29 August 2022). Up to ~45% of COVID-19 patients develop a mild-to-severe encephalopathy and encephalitis due to complications arising from viral-induced “***cytokine storm***,” elevated inflammatory signaling and/or anti-neural autoimmunity, sometimes referred to as “***cytokine storm syndrome***” (Mao et al., 2020; Vigasova et al., 2021; Baazaoui and Iqbal, 2022; Piekut et al., 2022). As is consistently observed in AD brain, the pro-inflammatory cytokines interleukin-1beta (IL-1β), IL-8, IL-18, the interleukin-1 receptor antagonist (IL-1RA) and serum neurofilament light (NF-L) chain protein, each a biomarker for all-cause pro-inflammatory neurodegeneration are positively associated with COVID-19 disease severity and are predictors of long-term outcome (Mao et al., 2020; Krey et al., 2021; Zetterberg and Schott, 2022). SARS-CoV-2 infected patients with existing AD are invariably associated with more severe complications of COVID-19 including increased morbidity and mortality (Mao et al., 2020; Krey et al., 2021; Chung et al., 2022; Guasp et al., 2022; Zetterberg and Schott, 2022). Depending upon COVID-19 disease course and post-infection severity multiple epidemiological studies indicate that about ~30–35% of all COVID-19 patients experience lasting neurological and neuropsychiatric symptoms ranging from relatively minor effects such as “***brain fog***” to more severe neurological complications. A pre-existing diagnosis of AD predicts the highest risk of COVID-19 infection yet identified, with the highest mortality among the most elderly AD patients (Song et al., 2021; Zhao et al., 2021; Ahmad et al., 2022; Baazaoui and Iqbal, 2022; Choe et al., 2022; Chung et al., 2022; Flud et al., 2022; Lingor et al., 2022; Visco et al., 2022). Interestingly, viral and/or other microbial infections, including SARS-CoV-2 invasion of the human brain and CNS, have long been known to contribute, intensify, propagate and/or augment the same neuropathological and pro-inflammatory neurodegenerative changes as is observed over the entire AD continuum from the earliest detectable forms of mild cognitive impairment (MCI) to the more severe terminal stages of AD (see below; Chiricosta et al., 2021; Ciaccio et al., 2021; Lingor et al., 2022; Lukiw et al., 2022; Piekut et al., 2022; Sirin et al., 2022; Szabo et al., 2022; Zhao and Lukiw, 2022).

## The SARS-CoV-2 “S1” spike protein, the ACE2R and amyloidogenesis

The SARS-CoV-2 virus possesses an unusually large, positive-sense single-stranded RNA (ssRNA) genome of about ~29,903 nucleotides (nt) packaged into a nucleocapsid core within a ~100 nm diameter virion particle that possesses a compact spherical lipoprotein envelope (SARS-CoV-2 isolate Wuhan-Hu-1, National Center for Biological Information (NCBI) GenBank Accession No. NC_045512.2; last accessed 29 August 2022; Ke et al., 2020; Sah et al., 2020; Wu et al., 2020; Mousavizadeh and Ghasemi, 2021; Zhao and Lukiw, 2022). Extending outward and decorating the surface of the SARS-CoV-2 lipoprotein envelope are the 672 amino acid homotrimeric ‘S1’ spike glycoproteins that play essential roles in ACE2R-recongition, viral attachment, fusion and entry into host cells to initiate SARS-CoV-2 infection (Duan et al., 2020; Ke et al., 2020; Zhao and Lukiw, 2022). Interestingly: **(i)** the ACE2R, normally a ubiquitously expressed zinc-containing metallo-carboxypeptidase (EC 3.4.17.23) surface receptor glycoprotein of the renin-angiotensin system (RAS) that has a role in the regulation of blood pressure is up-regulated in limbic regions of AD-affected brain (Ding et al., 2021; Zhao and Lukiw, 2022); **(ii)** the SARS-CoV-2 ‘S1’ spike protein is absolutely essential in ACE2R recognition and viral entry (Hill et al., 2021; Letarov et al., 2021; Palacios-Rápalo et al., 2021); **(iii)** the main antigen used as a target in COVID-19 vaccines is a lipid nanoparticle enclosing an RNA sequence encoding the full length SARS-CoV-2 ‘S1’ spike protein, since blocking ‘S1’ spike entry into host cells will prevent the initiation of SARS-CoV-2 infection (Actis et al., 2021; Dai and Gao, 2021); **(iv)** variations in the prion-like domains of the ‘S1’ spike protein differs among SARS-CoV-2 variants thus modulating ‘S1’ affinity for the ACE2R (Shahzad and Willcox, 2022; Tetz and Tetz, 2022); **(v)** SARS-CoV-2 ‘S1’ spike protein binds to the aggregation-prone glycosoaminoglycan heparin and heparin binding proteins (HBP) including amyloid-beta (Aβ) peptides, α-synuclein, tau and prion proteins and TDP-43 thus facilitating viral infection while accelerating the coalescence and aggregation of multiple pathological amyloidogenic proteins in the brain and CNS (Idrees and Kumar, 2021; Paiardi et al., 2022); **(vi**) targeting the interaction of SARS-CoV-2 ‘S1’ spike protein with these brain-enriched proteins may be a useful strategy to reduce pro-inflammatory aggregation processes that may limit the neurodegenerative disease process in COVID-19 patients (Clausen et al., 2020; Paiardi et al., 2022); and **(vii)** AD and COVID-19 infection share several important risk factors and comorbidities that include gender, aging, oxidative stress, hypertension, diabetes, APOE4 expression and up-regulation of the same families of inducible microRNAs (miRNAs), systemic inflammation and neuro-inflammation and/or the massive cytokine signaling disruptions referred to as the “***cytokine storm***” (Mao et al., 2020; Ciaccio et al., 2021; Vigasova et al., 2021). Interestingly just as the ACE2R is the most important cell surface receptor for SARS-CoV-2, elevated ACE2R expression appears to impose a significant risk factor for SARS-CoV-2 transmission in AD patients resulting in a higher viral load in AD-affected brain, and this may explain the high prevalence of SARS-CoV-2 infection among AD patients at any stage of the disease (Lim et al., 2020; Ding et al., 2021; Hill et al., 2021; Zhao et al., 2021; Shen et al., 2022; Zhao and Lukiw, 2022; Table 1).

**Table 1:** *Human neurological diseases and/or syndromes associated with ‘long COVID’*; recent reports of age-related, progressive, terminal and/or incapacitating neurological disorders associated with SARS-CoV-2 infection and COVID-19; the majority of these most recent reports involve COVID-19 with the neurodegenerative disorders AD, PrD (primarily Creutzfeldt-Jakob disease; CJD); and/or the onset of visual system disturbances (alphabetically ordered; Hill et al., 2021; Hixon et al., 2021; Oldfield et al., 2021; Zhou et al., 2021; Ahmad et al., 2022; Baazaoui and Iqbal, 2022; Flud et al., 2022; Lukiw, 2022; Piekut et al., 2022; Piras et al., 2022; Visco et al., 2022).

**Neurological disorder**	**Reference**
Alzheimer's disease (AD)	Hill et al., 2021
	Chiricosta et al., 2021
	Ciaccio et al., 2021
	Ding et al., 2021
	Zhao et al., 2021
	Shen et al., 2022
	Zhao and Lukiw, 2022
Epilepsy	Roy et al., 2021
Multiple sclerosis (MS)	Muñoz-Jurado et al., 2022
Prion disease (PrD)	Bernardini et al., 2022
	Ciolac et al., 2021
	Kuvandik et al., 2021
	Lukiw et al., 2022
	Olivo et al., 2022
	Shahzad and Willcox, 2022
	Szabo et al., 2022
	Tayyebi et al., 2022
	Tetz and Tetz, 2022
	Young et al., 2020
Visual system disturbances	Hill et al., 2021
	Hixon et al., 2021
	Tisdale et al., 2021
	Zhao et al., 2021
	Lukiw, 2022
	Piras et al., 2022

## SARS-CoV-2, prion neurobiology and prion disease

Human prion diseases (PrD) represent an expanding spectrum of progressive, and fatal neurodegenerative disorders affecting about one person in every one million per year worldwide, of which 80–95% are sporadic Creutzfeldt-Jacob disease (CJD) and the remainder representing genetic and/or familial CJD cases (Geschwind, 2015; Ayers et al., 2020). PrD infections are characterized by transmissibility, progressive neurological deficits caused by the accumulation of and aggregation of a misfolded “scrapie” isoform (PrPSc) from the native cellular prion protein (PrPc); and the rapid development of a progressive systemic inflammation very similar in nature to AD (Holmes et al., 2010; Ayers et al., 2020). A number of interesting associations are being made between SARS-CoV-2 infection and prion neurobiology and PrD: **(i)** several recent reports link multiple aspects of the ‘S1’ spike protein structure and function, immunology and epidemiology with PrD, prion-like spread and prion neurobiology (Letarov et al., 2021; Baazaoui and Iqbal, 2022; Paiardi et al., 2022; Shahzad and Willcox, 2022). Because ‘S1’ spike proteins support heparin and HBP interacions that promote the aggregation of Aβ peptides, α-synuclein, tau and prion proteins, SARS-CoV-2 infection itself may exacerbate the formation of amyloid peptide-enriched aggregates that support pro-inflammatory neurodegeneration, neuronal cell death and AD- and/or PrD-type change (Idrees and Kumar, 2021; Paiardi et al., 2022). ‘S1’ spike proteins containing ‘prion-like’ domains in free form may also play a role in systemic amyloidogenesis that in turn supports systemic inflammation, and the formation of pathogenic pro-inflammatory lesions in the brain and CNS (Letarov et al., 2021; Baazaoui and Iqbal, 2022; Shahzad and Willcox, 2022; Tetz and Tetz, 2022). Prion-like domains are known to self-associate, aggregate with other prion-like and HBP domains and amyloids, α-synuclein, tau and other prion proteins and contribute to protein-misfolding diseases that include AD and PrD infection (Holmes et al., 2010; Geschwind, 2015; Ayers et al., 2020); and **(ii)** there are several recent case studies of patients developing PrD and or exacerbating the neuropathology of PrDs such as CJD in conjunction with SARS-CoV-2 infection. Schmahmann's laboratory described a 60 yr old male patient whose first manifestations of CJD occurred in tandem with symptomatic onset of COVID-19. Quantification of a panel of the patient's systemic inflammatory mediators and biomarkers (including increased secretion of IL-1 and TNF) in response to the SARS-CoV-2-mediated-hastening of CJD pathogenesis suggested a significant relationship between host immune-responses to SARS-CoV-2 and an acceleration of inflammatory neurodegenerative cascades characteristic of CJD infection (Young et al., 2020). Olivo et al. described the case of a 70-year-old man with seizures and a rapidly evolving CJD during an acquired SARS-CoV-2 co-infection, again supporting the concept that CJD during SARS-CoV-2 infection is characterized by an accelerated progression of CJD (Olivo et al., 2022). Bernardini et al. (2022) recently described a ~40 year old male COVID-19 patient who developed CJD 2 months after COVID-19 onset with presenting symptoms of visuospatial deficits, hallucinations, ataxia and diffuse myoclonus-and their study concluded that the short interval between SARS-CoV-2 respiratory and CJD neurological symptoms was indicative of a causal relationship between a COVID-mediated neuroinflammatory state, protein misfolding and subsequent aggregation of PrPc into PrPSc, and emphasized the role of SARS-CoV-2 as an significant viral initiator of neurodegeneration (Bernardini et al., 2022). These developing molecularly- and clinically-evidenced associations between CJD and SARS-CoV-2 infection underscores an overlapping pathological link between PrD and COVID-19 both involving a systemic inflammation, a progressive and insidious lethal neurodegeneration and a potential acceleration of prion-like protein spread following SARS-CoV-2 viral invasion (Pogue and Lukiw, 2021; Song et al., 2021; Baazaoui and Iqbal, 2022).

Another interesting link between SARS-CoV-2 infection, PrD and the development of inflammatory neurodegeneration are the effects of these infections and their pathophysiological consequences on the abundance, speciation and complexity of a small family of inducible pathological microRNAs (miRNAs). These include, predominantly, the NF-kB (p50/p65)-sensitive miRNA-146a-5p and miRNA-155-5p and others (Zhao et al., 2020; Pogue and Lukiw, 2021; Pinacchio et al., 2022). A large amount of work has focused on the 22 nucleotide brain-enriched miRNA-146a-5p found to be significantly up-regulated in 10 known forms of PrD of both rodents and humans including CJD, in AD and in other sporadic and progressive age-related neurological disorders, and after infection by at least 18 neurotropic DNA and/or RNA viruses, including SARS-CoV-2, that infect the human brain, CNS, immune, lymphatic and hepatic, respiratory and/or circulatory systems (Pogue and Lukiw, 2021; Roganović, 2021; Pinacchio et al., 2022). Interestingly, the ACE2R recognized by the SARS-CoV-2 ‘S1’ spike protein is up-regulated by miRNA-146a and the many types of PrD and viral infections that induce miRNA-146a-5p and/or miRNA-155 are all associated with a advancing and insidious systemic inflammation and specific neurological disease symptoms and/or syndromes that are progressive, age-related, insidious, incapacitating and invariably fatal. Despite an apparent lack of nucleic acids in prions, both DNA- and RNA-containing viruses, along with prions, significantly and progressively induce miRNA-146a and/or miRNA-155 in the infected host, but whether this represents part of the host's adaptive immunity, innate-immune response or a mechanism to enable the invading prion or virus a successful infection remains incompletely understood (Ayers et al., 2020; Carlson and Prusiner, 2021; Pogue and Lukiw, 2021; Roganović, 2021; Pinacchio et al., 2022; Zhao and Lukiw, 2022).

## The multi-system and neurological impact of SARS-CoV-2 infection

It is important to emphasize that COVID-19 disease typically presents as an unusually rapid onset, highly transmissible viral pneumonia, and that SARS-CoV-2 infection initially requires a critical interaction between the viral ‘S1’ spike protein of SARS-CoV-2 and the surface membrane-exposed ACE2R. Some of the highest ACE2R densities have been found in the cholesterol- and sphingolipid-enriched lipid raft domains of multiple epithelial and endothelial cells of the human respiratory tract, however ACE2R has been identified on every human host cell type so far analyzed except for enucleated red blood cells (Hill et al., 2021; Palacios-Rápalo et al., 2021; Zhao et al., 2021; Kirtipal et al., 2022; Lukiw et al., 2022). ACE2R is abundantly detected in all cell types of the whole brain, CNS, neurovasculature, choroid plexus and the visual tracts extending from the retina to the occipital lobe that involve multiple visual processing and neuro-ophthalmic signaling pathways (Hill et al., 2021; Hixon et al., 2021; Zhao et al., 2021; Lukiw, 2022; Piras et al., 2022). Human vision and visual processing is negatively impacted by SARS-CoV2 infection (Hill et al., 2021; Hixon et al., 2021; Tisdale et al., 2021; Lukiw, 2022). Interestingly, the highest ACE2R expression yet described in the human CNS has been localized to the neurons of the medulla oblongata and pons in the brainstem, containing the Botzinger neuron complex and the brain's medullary respiratory centers, and this may in part explain the vulnerability of most SARS-CoV-2 infected patients to serious respiratory distress (Zhao et al., 2021; Lukiw et al., 2022; Molina-Molina and Hernández-Argudo, 2022). Besides the ubiquity and presence of the ACE2R on every human host cell type, all neural cell and tissue systems are linked together by a neural syncytium, a continuous intercellular networking system along which viruses may translocate (Kiyoshi and Zhou, 2019). Viruses such as SARS-CoV-2 also appear to utilize exosome- and vesicle-mediated transport mechanisms in systemic viral proliferation (Saheera et al., 2020; Eymieux et al., 2021; Visco et al., 2022). Using these various strategies for viral spread and means of translocation: **(i)** the SARS-CoV-2 virus has an enormous potential to infect, damage and/or destroy almost every cell, tissue type and organ system within the human host; and **(ii)** to induce a serious multi-organ system failure with highly interactive respiratory, cardiovascular, dermatologic, endocrine, gastrointestinal, hematologic, immunological, pulmonary, renal and/or neuro-ophthalmic, neurological or psychiatric complications across multiple human populations in diverse global environments (Mercatelli and Giorgi, 2020; Flud et al., 2022; Kirtipal et al., 2022; Rodriguez-Rivas et al., 2022; Visco et al., 2022).

## Discussion

It has been just over ~30 months since SARS-CoV-2 viral infection and COVID-19 disease were first described. SARS-CoV-2 infections are currently responsible for a serious and disturbing global pandemic in which just under ~600 million people have been infected and about ~6.5 million have died (https://www.worldometers.info/coronavirus/coronavirus-death-toll/; last accessed 29 August 2022). Over this relatively brief period of time about 40–60% of all “recovered” COVID-19 patients have experienced some type of ill-defined, wide-ranging and highly variable neurological complication and exhibit the symptomology of “long COVID.” Just as is the case for other incompletely characterized neurotrophic viral infections there are unexpected, unpredicted and sometimes alarming neurological and other sequelae to SARS-CoV-2-based viral infection. These include: **(i)** a pathological association with AD and novel onset human PrD; **(ii)** the recognition of self-associating prion-like viral domains in the SARS-CoV-2 ‘S1’ spike protein driving amyloidogenesis and neurotoxic aggregate formation; and **(iii)** the persistent emergence of novel SARS-CoV-2 viral strains highly resistant to natural host immune responses and the anti-‘S1’ spike glycoprotein-based vaccines and vaccination strategies (Oldfield et al., 2021; Bernardini et al., 2022; Rodriguez-Rivas et al., 2022; Shahzad and Willcox, 2022). Existing and ongoing research have uncovered significantly overlapping pathological neurology and neurochemistry and the involvement of multiple physiological systems in the complex and highly interactive disease mechanisms that define “***long COVID***,” PrD, neurodegeneration and SARS-CoV-2 neurobiology (Ritchie et al., 2020; Lingor et al., 2022; Lukiw et al., 2022; Olivo et al., 2022; Shahzad and Willcox, 2022; Visco et al., 2022). As in global pandemic infections of the past it is our opinion that we should anticipate additional unexpected associations of brain and CNS disease-linked mechanisms and pathways between SARS-CoV-2-mediated viral infection and other categories of age-related, immune-evasive pro-inflammatory forms of neurodegeneration. Importantly, the SARS-CoV-2 ‘S1’ spike proteins contain both self-associating “prion-like” regions, amyloid peptide-binding and other domains that appear to play roles in pathological “seeding,” amyloidogenesis and/or spreading that supports the formation of pathogenic lesions in the brain and CNS which contribute to pro-inflammatory neurodegeneration, neural cell atrophy and/or neuronal cell death (Tavassoly et al., 2020a,b; Lukiw et al., 2022; Tetz and Tetz, 2022).

The observed association between the more severe forms of COVID-19 and progressive neurodegenerative disorders that include AD and PrD at the molecular-genetic level are fascinating in that: **(i)** each disorder is a noteworthy example of a highly virulent, immune-evasive and often lethal neurotropic entity; and **(ii)** each of these pathogenic types are difficult to characterize, diagnose and treat, and possess unexpected characteristics, persistence and disease modalities (Baazaoui and Iqbal, 2022; Bernardini et al., 2022). The long-term effects and impact of COVID-19 disease infection and/or re-infection with the most recently identified SARS-CoV-2 strains including the SARS-CoV-2 ‘***Omicron stealth variants***’ BA.5 and/or B.1.1.529 (https://www.cdc.gov/coronavirus/2019-ncov/variants/variant-classifications.html; last accessed 29 August 2022) are not yet known. They are however sure to open new and intriguing chapters in the study of viral neurology, the epidemiology and neurobiology of these highly transmissible zoonotic entities, and multiple, highly interactive aspects of the human neurological and systemic pathophysiology associated with SARS-CoV-2 infection and neurodegeneration, especially in cases involving the elderly and in immunologically compromised human populations.

## Author contributions

YZ, VJ, and WL collected, analyzed, summarized the literature, and formulated an overall opinion on this contemporary topic. WL wrote the article. All authors contributed to the article and approved the submitted version.

## Funding

Research on viral infection of the human brain with focus on HSV-1, SARS-CoV-2 and prion disease, single-stranded RNA (ssRNA) including SARS-CoV-2, miRNA, miRNA-mRNA and ssRNA-mRNA interactions involving neuronal atrophy, altered synaptic signaling, AD innate-immune response and neuro-inflammation was supported through Translational Research Initiative Grants from LSUHSC, The Brown Foundation, Joe and Dorothy Dorsett Innovation in Science Healthy Aging Award and NIA Grants AG18031 and AG038834.

## Conflict of interest

The authors declare that the research was conducted in the absence of any commercial or financial relationships that could be construed as a potential conflict of interest.

## Publisher's note

All claims expressed in this article are solely those of the authors and do not necessarily represent those of their affiliated organizations, or those of the publisher, the editors and the reviewers. Any product that may be evaluated in this article, or claim that may be made by its manufacturer, is not guaranteed or endorsed by the publisher.
